# Effect of fenofibrate and selective PPARα modulator (SPPARMα), pemafibrate on KATP channel activity and insulin secretion

**DOI:** 10.1186/s13104-023-06489-7

**Published:** 2023-09-11

**Authors:** Shigeki Kitamura, Naoya Murao, Shoko Yokota, Masaru Shimizu, Tomoyuki Ono, Yusuke Seino, Atsushi Suzuki, Yuko Maejima, Kenju Shimomura

**Affiliations:** 1 Department of Bioregulation and Pharmacological Medicine, Fukushima Medical University School of Medicine, 1 Hikarigaoka, Fukushima, 960-1295 Japan; 2 Department of Plastic and Reconstructive Surgery, Fukushima Medical University School of Medicine, Fukushima, Japan; 3 Department of Endocrinology, Diabetes and Metabolism, Fujita Health University, Toyoake, Japan; 4 Department of Neurology, Matsumura General Hospital, Iwaki, Japan

**Keywords:** KATP channel, PPARα, fenofibrate, Pemafibrate

## Abstract

**Objective:**

Insulin secretion is regulated by ATP-sensitive potassium (K_ATP_) channels in pancreatic beta-cells. Peroxisome proliferator-activated receptors (PPAR) α ligands are clinically used to treat dyslipidemia. A PPARα ligand, fenofibrate, and PPARγ ligands troglitazone and 15-deoxy-∆^12,14^-prostaglandin J2 are known to close K_ATP_ channels and induce insulin secretion. The recently developed PPARα ligand, pemafibrate, became a new entry for treating dyslipidemia. Because pemafibrate is reported to improve glucose intolerance in mice treated with a high fat diet and a novel selective PPARα modulator, it may affect K_ATP_ channels or insulin secretion.

**Results:**

The effect of fenofibrate (100 µM) and pemafibrate (100 µM) on insulin secretion from MIN6 cells was measured by using batch incubation for 10 and 60 min in low (2 mM) and high (10 mM) glucose conditions. The application of fenofibrate for 10 min significantly increased insulin secretion in low glucose conditions. Pemafibrate failed to increase insulin secretion in all of the conditions experimented in this study. The K_ATP_ channel activity was measured by using whole-cell patch clamp technique. Although fenofibrate (100 µM) reduced the K_ATP_ channel current, the same concentration of pemafibrate had no effect. Both fenofibrate and pemafibrate had no effect on insulin mRNA expression.

## Introduction

Diabetes and dyslipidemia are two major global health concerns; they both have strong associations with life threatening ischemic strokes [1].

Clinically, fibrates are used to treat dyslipidemia. In the Action to Control Cardiovascular Risk in Diabetes (ACCORD)-Lipid trial, the major fibrate drug, fenofibrate, improved cardiovascular disease outcomes in high triglyceride (TG) patients [2]. Fibrates are ligands of the nuclear receptor peroxisome proliferator-activated receptor (PPAR) α. PPAR is divided into three subtypes, α, β and γ [3]. PPARα is known to regulate fatty acid metabolism while γ is known to involve in glucose homeostasis and adipocyte proliferation [4, 5].

Recently, a new PPARα ligand, pemafibrate, was developed and used in the clinical setting for the treatment of dyslipidemia. Pemafibrate has high selectivity to PPARα with greater activation capability compared to other fibrate drugs and is classified as a selective PPARα modulator (SPPARMα) [6]. Clinically, pemafibrate has also been reported to have a significantly greater effect on decreasing TG levels compared to fenofibrate [7]. In addition to fatty acid regulation, there are reports indicating that PPARα ligands affect insulin secretion. Sun et al. and Dong et al. reported that PPARα ligand enhanced glucose-stimulated insulin secretion from isolated rodent islet and beta cell line INS-1 cells [8, 9].

Insulin secretion is stimulated by the closure of ATP-sensitive K^+^ (K_ATP_) channels in pancreatic beta cells. An increase of intracellular ATP induced by the glucose metabolism closes K_ATP_ channels [10, 11]. The closure of K_ATP_ channels leads to membrane depolarization and opening of voltage dependent Ca^2+^ channels, which allows Ca^2+^ influx, ultimately leading to insulin release [10].

Sulfonylureas and glinides, such as glibenclamide and repaglinide, close K_ATP_ channels and induce insulin secretion; thus, they are used to treat diabetic patients [12, 13].

We previously reported that a PPARα ligand, fenofibrate, and PPARγ ligands, troglitazone and 15-deoxy-∆^12,14^-prostaglandin J2, directly interact with and close K_ATP_ channels and induce insulin secretion in pancreatic beta cell line HIT-T15 cells [14]. Since these PPAR ligands were able to close K_ATP_ channels and induce insulin secretion, it is possible that SPPARMα ligand pemafibrate may also close K_ATP_ channels and induce insulin secretion. Since pemafibrate is widely used clinically, it is important to confirm this possibility.

Here we investigated the effects of pemafibrate, and those of fenofibrate, on K_ATP_ channel activity and insulin secretion.

## Materials and methods

### Insulin secretion

MIN6 cells, kindly provided by Prof Susumu Seino at Kobe Univ [15], were plated in 6-multiwell plates (1 × 10^5^ cells per well) cultured with high-glucose DMEM medium containing 10% heat-inactivated FBS in a humidified incubator with 95% O_2_ and 5% CO_2_ at 37 ℃. On the day of the experiment, the cells were starved for 1 h in 2 mM glucose solution and replaced with 2 ml of experimental medium and insulin secretion was measured by static incubation (10 min and 60 min). The experimental media were based on the Krebs–Ringer buffer. The Krebs–Ringer buffer contained (in mM) 118.5 NaCl, 2.54 CaCl, 1.19 KH_2_PO_4_, 4.74 KCl, 25 NaHCO_3_, 1.19 MgSO_4_, and 10 HEPES (pH 7.4 with NaOH) with 0.1% bovine serum albumin. Insulin was measured using ELISA assay kit (Cat No. M1104, Morinaga, Yokohama, Japan). Fenofibrate was purchased from Sigma (Cat No. F6020, St. Louis, MO, USA). Pemafibrate was kindly provided by Kowa Co. Ltd (Nagoya, Japan). 100 mM stock solutions of pemafibrate and fenofibrate were prepared in DMSO and used in the experiment by diluting 1000 × to acquire 100 μM concentration. All control samples contained the same amount of DMSO.

### Electrophysiology

Electrophysiological experiments were performed as previously described [16–18]. All electrophysiological measurements were performed at room temperature (22–25 ℃) using an EPC-800 patch-clamp amplifier (HEKA, Lambrecht/Pfalz, Germany) and pCLAMP 10 software (Molecular Devices, CA, USA). The pipette solution contained (in mM) 107 KCl, 2 MgCl_2_, 1 CaCl_2_, 10 EGTA, 10 HEPES, and 0.3 ATP (pH 7.2 with KOH), and the extracellular solution contained (in mM) 138 NaCl, 5.6 KCl, 1 MgCl_2_, 10 HEPES, and 2.6 CaCl_2_ (pH 7.4 with NaOH). The effects of 100 µM of fenofibrate and pemafibrate on K_ATP_ channel currents were evaluated using the standard whole-cell technique by applying a holding potential of − 70 mV with ± 10 mV steps at a duration of 250 ms. Data were analyzed using Clampfit software (Molecular Devices).

### Reverse transcription-quantitative polymerase chain reaction (qRT-PCR) analysis

The MIN6 cells were exposed to 100 µM fenofibrate or pemafibrate for 2 h. Following the application of drugs, total RNA was isolated using a RNeasy minikit (Cat No. 74104, QIAGEN, Hilden, Germany) and Monarch RNA Purification Columns (Cat No. T2007, New England BioLabs Japan, Inc., Massachusetts, USA). c-DNA synthesis was performed using M-MLV (Cat No. 28025013, Thermo Fisher Scientific, Massachusetts, USA), RNaseOUT Recombinant Ribonuclease Inhibitor (Cat No. 10777019. Thermo Fisher Scientific, Massachusetts, USA), and dNTP (Cat No. 200415, Agilent Technologies, Texas, USA). A quantitative RT-PCR assay was performed using the TB Green Premix Ex Taq II (Tli RNaseH Plus, Cat No. RR820, Takara Bio Inc., Shiga, Japan). The cycling condition was as follows: initial denaturation at 95 ℃ for 30s, then 40 cycles each at 95 ℃ for 5s, 56 ℃ for 10s, and 72 ℃ for 15s, according to the protocol. Product accumulation was measured in real time and the mean cycle thresholds were determined. The expression levels of Ins1 and Ins2 were calculated using the 2^ΔΔCT^ method of relative quantification and normalized by the housekeeping gene glyceraldehyde-3-phosphate dehydrogenase (GAPDH). The PCR primers are as follows: Ins1 (NM_008386): Fw (CCAGCTATAATCAGAGACCA), Rev (GGGCCTTAGTTGCAGTAGTT), Ins2 (NM_001185083): Fw (AGCGTGGCTTCTTCTACACAC), Rev (CTGGTGCAGCACTGATCTACA), GAPDH (NM_001289726): Fw (TCCACTCACGGCAAATTCAACG), Rev (TAGACTCCACGACATACTCAGC).

### Statistical analysis

All data are expressed as means ± SEM. The statistical significance of differences was assessed using a paired t-test for electrophysiological changes in currents and one-way ANOVA followed by Tukey’s test for insulin secretion and qRT-PCR results. P < 0.05 was considered as significant difference.

## Results

### Effect of pemafibrate and fenofibrate on insulin secretion from pancreatic beta-cell line

**Fig. 1:**
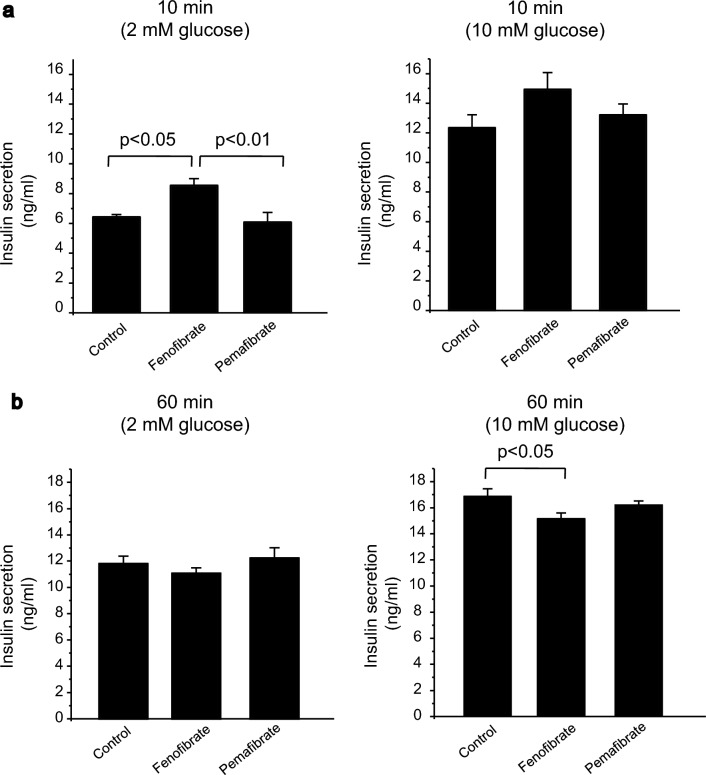
The effect on insulin secretion of fenofibrate and pemafibrate on MIN6 cells. **a** Insulin secretion from MIN6 cells for 10 min incubation of control, pemafibrate (100 µM), and fenofibrate (100 µM) in 2 mM glucose (left) and 10 mM glucose (right) conditions (n = 6 independent wells in each condition). **b** Insulin secretion from MIN6 cells for 60 min incubation of control, pemafibrate (100 µM), and fenofibrate (100 µM) in 2 mM glucose (left) and 10 mM glucose (right) conditions (n = 6 independent wells in each condition)

First, we measured the insulin secretion from cultured pancreatic beta-cell line MIN6 cells in various conditions. The 10 min application of fenofibrate in low glucose conditions (2 mM glucose) significantly increased insulin secretion compared to the control and pemafibrate (Fig. 1a, left). Pemafibrate application for 10 min did not induce an increase in insulin secretion compared to the control. The amounts of insulin secreted were 6.45 ± 0.14 ng/ml for the control, 8.56 ± 0.45 ng/ml for fenofibrate, and 6.08 ± 0.64 ng/ml for pemafibrate. In high glucose conditions (10 mM glucose), the 10 min application of fenofibrate tended to increase insulin secretion, but the increase was not statistically significant compared to the control or pemafibrate (Fig. 1A, right). The amounts of secreted insulin were 12.36 ± 0.86 ng/ml for the control, 14.94 ± 1.14 ng/ml for fenofibrate, and 13.23 ± 0.73 ng/ml for pemafibrate.

In long term applications, both fenofibrate and pemafibrate did not show significant differences in insulin secretion compared to the control in low glucose conditions (11.84 ± 0.57 ng/ml for the control, 11.10 ± 0.41 ng/ml for fenofibrate, and 12.26 ± 0.79 ng/ml for pemafibrate) (Fig. 1b, left). However, in high glucose conditions, fenofibrate showed a significant decrease in insulin secretion (16.77 ± 0.58 ng/ml for the control, 15.07 ± 0.43 ng/ml for fenofibrate, and 16.11 ± 0.32 ng/ml for pemafibrate) (Fig. 1b, right).

### Effect of fenofibrate and pemafibrate on KATP channel activity

We recorded the K_ATP_ channel current of MIN6 cells using the whole-cell patch-clamp technique. The application of 20 mM glucose did not affect K_ATP_ channel currents, indicating that the intracellular complex, such as the glycolysis system, is replaced by a pipette solution.

**Fig. 2:**
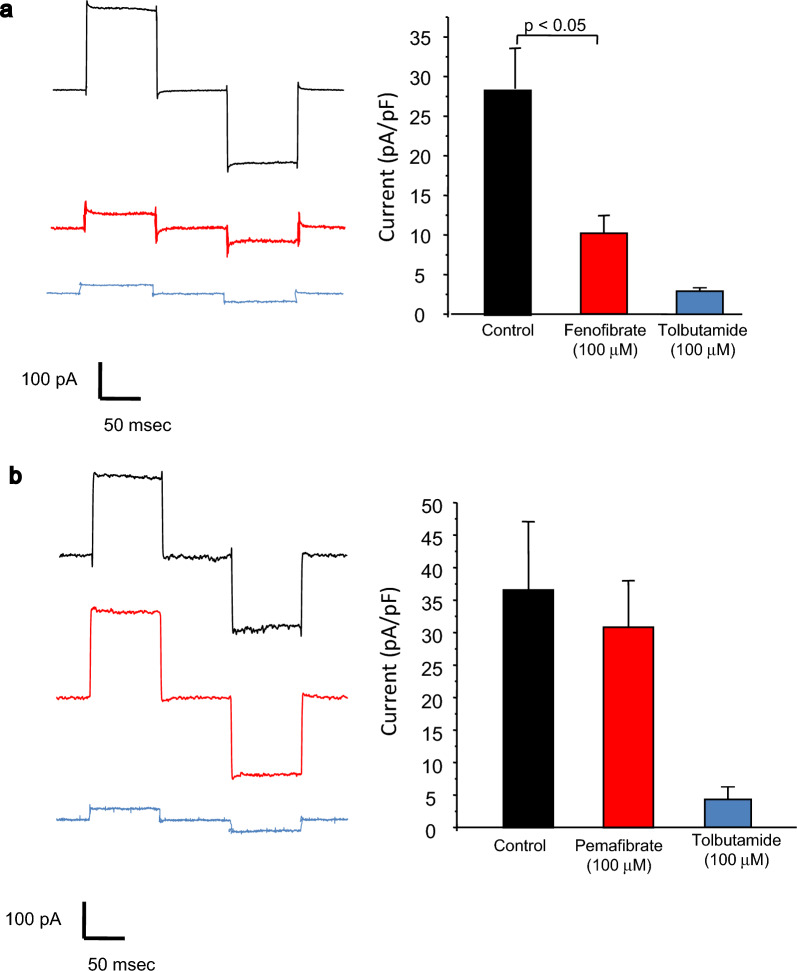
Effect of fenofibrate and pemafibrate on K_ATP_ channel activity. **a** left: Representative K_ATP_ channel recordings from MIN6 cells before (black), after application of 100 µM fenofibrate (red) and after application of 100 µM tolbutamide (blue) during the holding potential of − 70 mV with ± 10 mV steps at a duration of 250 ms; right: Bar graph showing the summary of K_ATP_ channel currents of MIN6 cells before (black),after application of fenofibrate (red) and after application of tolbutamide (blue). n = 10 cells. **b,** left: Representative K_ATP_ channel recordings from MIN6 cells before (black), after application of 100 µM pemafibrate (red) and after application of 100 µM tolbutamide (blue) during the holding potential of − 70 mV with ± 10 mV steps at a duration of 250 ms; right: Bar graph showing the summary of K_ATP_ channel currents of MIN6 cells before (black), after application of pemafibrate (red) and after application of tolbutamide (blue). n = 10 cells

Consistent with the results of our previous study, the application of 100 µM fenofibrate reduced K_ATP_ channels in MIN6 cells (Fig. 2A). The K_ATP_ channel current before the application of fenofibrate (the control) was 28.46 ± 5.11 pA/pF, while after the application of fenofibrate it was 10.27 ± 1.78 pA/pF. The application of 100 µM pemafibrate showed no effect on the K_ATP_ channel current (Fig. 2b). The K_ATP_ channel current before the application of pemafibrate (the control) was 36.66 ± 10.46 pA/pF, while after the application of pemafibrate it was 30.94 ± 7.11 pA/pF. The current recorded in this study was confirmed to be the K_ATP_ channel current by applying a selective blocker of the K_ATP_ channel (100 µM tolbutamide).

### Effect of fenofibrate and pemafibrate on insulin mRNA expression in MIN6 cells

**Fig. 3:**
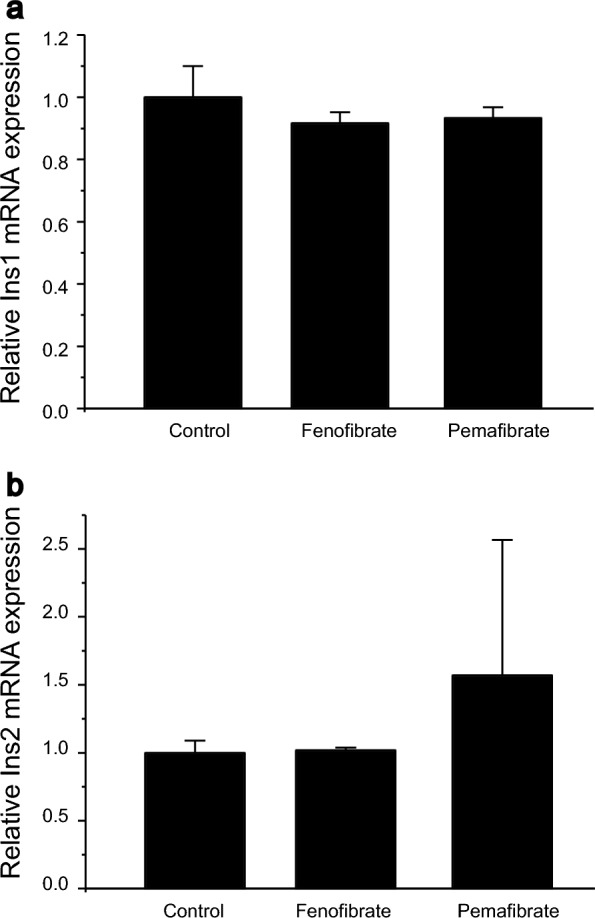
Relative expression of insulin mRNA expression with fenofibrate and pemafibrate application. **a** Relative mRNA expression of Ins1 mRNA control, and after 2 h fenofibrate (100 µM) and pemafibrate (100 µM) application on MIN6 cells. n = 5 each from independent experiment. **B** relative mRNA expression of Ins2 mRNA control, and after 2 h fenofibrate (100 µM) and pemafibrate (100 µM) application on MIN6 cells. n = 5 each from independent experiment

Because fenofibrate showed a significant reduction of insulin secretion in long term application under high glucose conditions, we measured the influence of these two drugs on insulin gene expression. No difference in Ins1 or Ins2 mRNA expression was confirmed after 100 µM of fenofibrate or pemafibrate applications (Fig. 3a, b).

## Discussion

In the present study, we showed that SPPARMα pemafibrate has no direct effect on insulin secretion, whereas the PPARα ligand fenofibrate blocks K_ATP_ channels, increases short term insulin secretion in low glucose conditions, and reduces long term insulin secretion in high glucose conditions.

PPARα ligands are reported to have glucose-lowering effects in type 2 diabetic patients and diabetic model mice [8, 19, 20]. The underlying mechanism for lowering glucose is considered to be the ligands acting on both insulin sensitivity and pancreatic beta-cells. Regarding the effect on insulin sensitivity, PPARα ligands are reported to increase TG and fatty acid metabolism, thus reducing the fatty acid contents in tissues such as those in the liver and skeletal muscle [21, 22]. In addition, suppression of inflammatory cytokine production from monocytes is also considered to be a mechanism of improving insulin sensitivity [23]. Regarding the effects of PPARα ligands on pancreatic beta-cells, fenofibrate is reported to potentiate glucose-stimulated insulin secretion (GSIS) under high palmitate conditions [8]. Pemafibrate is also reported to improve insulin secretion by increasing the expression of ATP-binding cassette protein A1 (ABCA1), which is a critical regulator of cholesterol and phospholipid efflux [9]. It is likely that PPARα ligands indirectly improve pancreatic beta-cell function by ameliorating lipotoxicity. However, reports are showing PPARα ligands stimulating insulin secretion by directly acting on beta-cell function. Pemafibrate is reported to ameliorate oxidative stress of pancreatic beta-cells [24]. Since the expression of antioxidant enzymes are low in pancreatic beta-cells [25], the effect of pemafibrate on reducing oxidative stress is more in the maintaining of beta-cell condition rather than the insulin secretion process. However, we have shown in the past that fenofibrate directly affects the insulin secretion process by closing K_ATP_ channels.

The K_ATP_ channels are a key factor in regulating insulin secretion in pancreatic beta-cells. The ATP produced from glucose metabolism directly interacts with, and closes, K_ATP_ channels, and induces insulin secretion. Reduction of ATP sensitivity in K_ATP_ channels induces a reduction in insulin secretion. The reduction of ATP sensitivity of K_ATP_ channels due to mutation can cause a special type of diabetes known as neonatal diabetes [26, 27].

In the present study, fenofibrate significantly increased insulin secretion in short term applications in low glucose conditions, and only tended to increase under high glucose conditions. This may be explained in that the increase of intracellular ATP reflecting high glucose conditions may overcome the inhibitory effect of fenofibrate on K_ATP_ channels. Further study of fenofibrate on K_ATP_ channel kinetics is required to elucidate the details of the mechanism for fenofibrate blocking the channels.

**Fig. 4:**
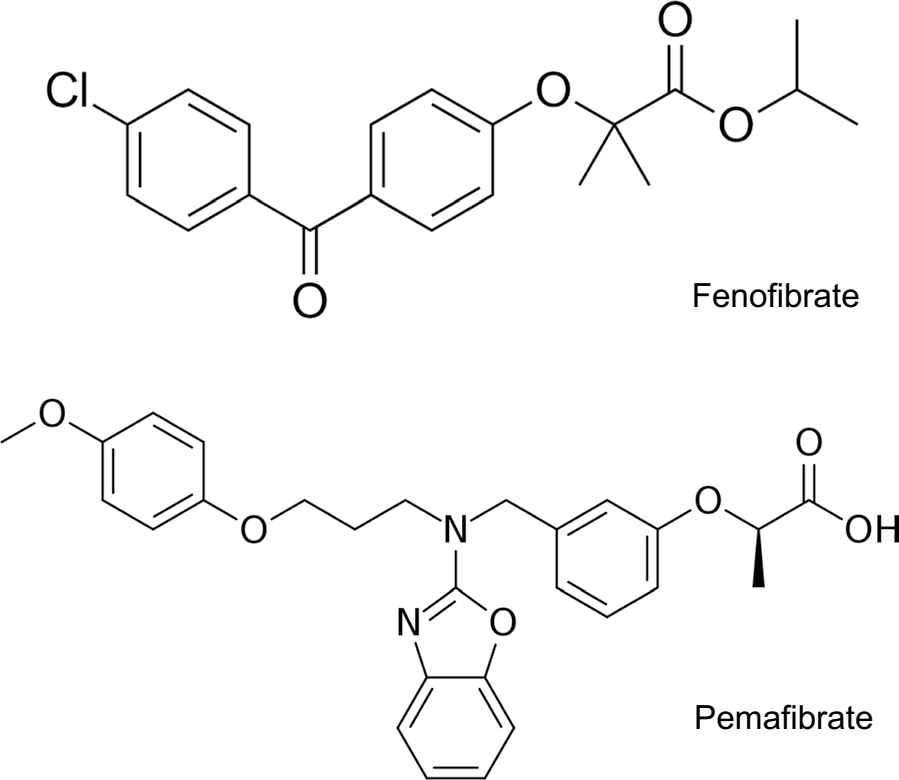
Structure of fenofibrate and pemafibrate

To date, there are no reports of hypoglycemia from patients under fenofibrate treatment. This is because fenofibrate is rapidly converted into fenofibric acid, the pharmacologically relevant form of PPARα, in liver and plasma [28]. However, we have shown in our previous study that fenofibric acid also closes K_ATP_ channels and induces insulin secretion in high doses. In addition, PPARγ agonists, such as troglitazone and 14-deoxy ∆^12,14^-PGJ_2_, have been confirmed to close K_ATP_ channels and induce insulin secretion [14]. Therefore, with pemafibrate being a highly specific ligand of PPARα and fibrate class drug, it is important to confirm whether it affects K_ATP_ channels and insulin secretion similar to fenofibrate or PPARγ ligands. The present study clearly shows that pemafibrate does not affect K_ATP_ channel activity, insulin secretion, or insulin gene expression. Structurally, fenofibrate and pemafibrate have a common acidic region but pemafibate uniquely contains benzoxadole and phenoxyalkyl side chains (Fig. 4). These structural differences may contribute to different effects on insulin secretion and K_ATP_ channel activity.

Although pemafibrate showed no effect on insulin secretion, we have shown in this study that 60 min applications of fenofibrate can significantly reduce insulin secretion compared to the control in high glucose conditions. In the past, Ramarkrishnan et al. reported that mice treated with fenofibrate for 4 weeks showed a reduction in insulin secretion [29]. The underlying mechanism for this in vivo effect of a reduction of insulin secretion induced by fenofibrate treatment was clarified to be a mechanism compensating for the increased plasma insulin level due to the fenofibrate’s PPARα activation-triggered reduction of insulin clearance in the liver. In the same report, it was further confirmed that in vitro experiments using islets isolated from the same mice did not show an increase in insulin secretion. This is in direct contrast to the findings of the present study. Because insulin mRNA expression was not affected by the fenofibrate in our study, the underlying mechanism for our results may not involve the transcriptional activity of insulin genes. The fact that this effect was only observed in high glucose conditions may indicate the involvement of glucose metabolism. Further studies are required.

## Conclusions

While fenofibrate can inhibit the K_ATP_ channel and induce a significant increase of insulin secretion in low glucose concentration within 10 min, pemafibrate showed no such effect. It indicates that when these drugs are clinically used to treat dyslipidemia, fenofibrate may increase insulin secretion while pemafibrate has no such effect. In addition to the pharmacological actions we have shown in this study, our current findings contain important information on possible additional effects and pharmacological mechanism of PPARα ligands. Clinically, the findings of the present study that suggest the possible involvement of PPARα ligands in insulin secretion provide new information for the treatment of diabetes as well.

## Data Availability

The data that support the findings of this study are available from the corresponding author upon reasonable request.

## Abbreviations

K_ATP_ channel: ATP sensitive potassium channel
PPAR: Peroxisome proliferator-activated receptors selective
SPPARα: PPARα modulator
